# Prevalence and Predictors of Vitamin D Insufficiency in Children: A Great Britain Population Based Study

**DOI:** 10.1371/journal.pone.0022179

**Published:** 2011-07-22

**Authors:** Michael Absoud, Carole Cummins, Ming J. Lim, Evangeline Wassmer, Nick Shaw

**Affiliations:** 1 School of Health and Population Sciences, University of Birmingham, Birmingham, United Kingdom; 2 Department of Paediatric Neurology, Evelina Children's Hospital at Guy's and St Thomas' NHS Foundation Trust, London, United Kingdom; 3 Department of Paediatric Neurology, Birmingham Children's Hospital NHS Foundation Trust, Birmingham, United Kingdom; 4 Department of Paediatric Endocrinology, Birmingham Children's Hospital NHS Foundation Trust, Birmingham, United Kingdom; Pennington Biomedical Research Center, United States of America

## Abstract

**Objectives:**

To evaluate the prevalence and predictors of vitamin D insufficiency (VDI) in children In Great Britain.

**Design:**

A nationally representative cross-sectional study survey of children (1102) aged 4–18 years (999 white, 570 male) living in private households (January 1997–1998). Interventions provided information about dietary habits, physical activity, socio-demographics, and blood sample. Outcome measures were vitamin D insufficiency (<50 nmol/L).

**Results:**

Vitamin D levels (mean = 62.1 nmol/L, 95%CI 60.4–63.7) were insufficient in 35%, and decreased with age in both sexes (p<0.001). Young People living between 53–59 degrees latitude had lower levels (compared with 50–53 degrees, p = 0.045). Dietary intake and gender had no effect on vitamin D status. A logistic regression model showed increased risk of VDI in the following: adolescents (14–18 years old), odds ratio (OR) = 3.6 (95%CI 1.8–7.2) compared with younger children (4–8 years); non white children (OR = 37 [95%CI 15–90]); blood levels taken December-May (OR = 6.5 [95%CI 4.3–10.1]); on income support (OR = 2.2 [95%CI 1.3–3.9]); not taking vitamin D supplementation (OR = 3.7 [95%CI 1.4–9.8]); being overweight (OR 1.6 [95%CI 1.0–2.5]); <1/2 hour outdoor exercise/day/week (OR = 1.5 [95%CI 1.0–2.3]); watched >2.5 hours of TV/day/week (OR = 1.6[95%CI 1.0–2.4]).

**Conclusion:**

We confirm a previously under-recognised risk of VDI in adolescents. The marked higher risk for VDI in non-white children suggests they should be targeted in any preventative strategies. The association of higher risk of VDI among children who exercised less outdoors, watched more TV and were overweight highlights potentially modifiable risk factors. Clearer guidelines and an increased awareness especially in adolescents are needed, as there are no recommendations for vitamin D supplementation in older children.

## Introduction

### Background

Vitamin D functions as a hormone and its importance in the immunomodulatory effects on disease is being increasingly recognised [1], [2]. In the UK, there is evidence of a resurgence of vitamin D deficiency in children [3], [4], [5], [6]. The National Diet and Nutrition Survey (NDNS) for young people [7] (4–18 years old) in Great Britain has already reported that approximately 8% of children had 25-hydroxy Vitamin D (25[OH]D) levels <25 nmol/L. Data published from the NDNS has also shown that there is evidence of Vitamin D deficiency (<25 nmol/L) in most population age groups including in older children and young adults [8], [9]. In a US study of vitamin D levels of children aged 1–11 years old, 18% had levels of <25 nmol/L [10]. Factors known to influence vitamin D metabolism include skin pigmentation, age, ethnicity, sunshine exposure, body mass index, and season. Dietary factors include intake of vitamin D rich food, vitamin D supplementation, and the association of obesity with lower vitamin D levels [8], [11]. Genetic factors have also recently been implicated in the regulation of vitamin D status [12], and its association with chronic diseases such as multiple sclerosis, hyperglycaemia and the metabolic syndrome, hypertension, type 1 diabetes, and malignancy [2], [13], [14]. Recent UK government published guidelines [15] recommend vitamin D supplementation (7 micrograms/day) only to children younger than 5 years of age (Healthy Start initiative). However, recommendations have generally not been implemented as vitamins have only been made freely available to those receiving specific government benefits [8], [16]. There are no UK Reference Nutrient Intake values for vitamin D or government supplementation recommendations for older children; although the 2007 Scientific Advisory Committee on Nutrition report [8] does recommend that individuals who are at risk of inadequate sunshine exposure should receive supplementation (10 micrograms/day).” Additionally the government recommendations advise regular, short periods of sunlight exposure without sunscreen during the summer months, they also highlight the risk of skin cancer and recommend the use of sunscreen (with a high UVB factor) for the majority of time spent outside.

There is increasing consensus that vitamin D insufficiency (VDI) should be defined as levels of <50 nmol/L [16], [17], [18]. There is a paucity of data in the UK and indeed worldwide on the prevalence, predictors and associations of VDI. There have been three recent and relatively large cross-sectional studies exploring associations with vitamin D deficiency and associations: (1) National Health and Nutrition Examination Survey (NHANES) in the USA [19] (n = 6275), Philadelphia [20] (n = 382) and New Zealand [21] (n = 144). However, the studies were either single site with Vitamin D data available for a subset of cases (Philadelphia), included a narrow age range (12–22 months) of patients (New Zealand) or unable to evaluate specific important predictors (and or determinants) of VDI such as season of measurement of 25(OH)D levels, the latitude of the participants' residents, and information on outdoor play and sports from the NHANES study.

Additionally, there is also a paucity of data on the relative importance of proposed predictors for vitamin D status in UK children [16]. There is a need to identify factors that need to be adequately controlled for when conducting case control studies involving vitamin D and its association with autoimmune conditions. Furthermore, clinicians need to be better informed as to when to recommend vitamin D supplementation for children. Finally, it is important to characterise the relative importance of modifiable risk factors, as this will inform wider recommendations concerning the prevention of VDI.

### Objective

The aim of this study was to describe vitamin D status and comprehensively evaluate important currently accepted predictors of VDI in Great Britain, in children aged 4–18 years of age by examining data obtained from the NDNS [22] of young people.

## Methods

### Study Design

The survey plan and procedures have been described in detail in the NDNS report [7] (Gregory et al, 2000). Briefly, a nationally representative sample of young people aged 4 to 18 years living in private households was acquired. Fieldwork covered a 12 month period (January 1997–1998), to cover any seasonality of factors related to nutritional status. Where there was more than one young person living in the same household, only one was randomly selected to take part in the survey. The sample was selected using a multi-stage random probability design with postal sectors as first stage units. The elements of the survey included:

- a detailed interview to provide general information about dietary habits and background information about lifestyle and socio-demographic characteristics;
- a seven-day weighed intake dietary record of food and drink consumed;
- a seven-day physical activity diary for young people aged 7–18 years;
- physical measurements — height and weight;
- a fasting blood sample (with written consent) analysed. This sample was subdivided and used for the measurement of a wide range of biochemical status analyses in three laboratories.

Children aged 11 years and older were expected to keep their own dietary and physical activity record. Younger children needed varying levels of help from parents, teachers and other carers. To help the nutritionists evaluate the quality of the dietary records completed by the young people, interviewers completed a quality assessment questionnaire. This included information on how accurate the interviewers thought the weighing and recording of items eaten had been and whether the diary was an accurate reflection of the young person's actual diet. In a sub-group of a feasibility study sample the validity of the dietary recording methodology was tested using the doubly-labelled water methodology to compare energy expenditure against reported energy intake [7]. For the same sub-group the physical activity information collected in the diary was validated by directly measuring the young person's activity level using a motion sensor.

### Vitamin D assay

The NDNS currently uses the DiaSorin Liaison Total assay (formerly Incstar Minnesota, USA) for the measurement of 25(OH)D. There were 800 micro-litres of serum available for 25(OH)D analysis. The radioimmunoassay kit which was used was based on the developmental work by Hollis et al [23]. The Vitamin D assay was conducted at the Medical Research Council (MRC) Dunn Nutrition Unit (DNU) in Cambridge, UK (now the MRC Human Nutrition Research). Analysis of samples was conducted in a sequence so as to avoid bunching within fieldwork areas, during each batch analysis. Each assay was performed in duplicate, and if agreement between duplicates failed to meet quality controls repeat assays were performed. Percentage coefficient of variation reported for the 4 fieldworks was 15.38–21.11% [7]. In addition, 20 independent samples for inter-laboratory quality assurance comparisons were analysed yielding a mean deviation of 0.02 for sample concentration 12.9–81.4 nM.

### Participants and sample size

Data from the NDNS [22] of young people aged 4–18 years were utilised as the survey is the largest and most detailed ever undertaken of the diet and nutritional status of young people in Britain. The NDNS programme aims to provide a comprehensive, cross-sectional picture of the dietary habits and nutritional status of the population of Great Britain living in private households. The multivariable nature of prognostic research makes it difficult to estimate required sample size, and ideally require several hundred outcome events [24]. The NDNS provides a suitable sample as a total of 1102 Vitamin D (25[OH]D) blood sample results (from a total of 1701 subjects interviewed) were available for analysis, and of those 917 had detailed interview, and physical activity diary records.

Weighting factor adjustments were used to adjust for known socio-demographic differences, and differential probability for having blood tests between the composition of the survey sample and that of the entire (census) population of Great Britain.

### Variables used as predictors

Proposed candidate predictors which have previously been reported as prognostic were used. The following variables were derived from the dataset and investigated as predictors:

- Age, gender and ethnicity
- Whether on income Support
- Region residing
- Month blood was taken
- Amount spent per week in sports involving outdoor exercise or play
- Amount spent a day per week watching TV
- Vitamin D dietary intake and supplemental Vitamin D intake
- Body Mass Index (BMI [kg/m^2^])

To classify overweight and obesity in young people of 2–18 years of age, cut offs described by Cole et al (on behalf of the International Obesity Task Force) were used [25], [26]. Cole et al developed a series of age and sex-specific BMI cut-off points based on data collected from UK, Brazil, Hong Kong, Singapore, the Netherlands, and the USA. These BMI cut-offs were derived from sex specific BMI age curves that pass through a BMI of 25 and 30 (the cut-off points used in adults to define overweight and obesity) at 18 years of age, respectively.

### Outcomes measured

For the purposes of this study, Vitamin D status was defined [16] as insufficient when 25(OH)D levels were <50 nmol/L).

### Statistical methods

Statistical analysis was carried out with SPSS version 17.0 and included descriptive statistics, univariate regression and binary loglinear regression. In the different models, Vitamin D was used as a continuous variable, and as a binary outcome for insufficiency (<50 nmol/L). Chi squared and t-tests were also used to investigate significance of proposed predictors for VDI and vitamin D status respectively. For the binary logistic regression (for VDI), the dataset was split into two parts (70% and 30%) and the model developed on the larger sample called the ‘training dataset.’ A backward elimination approach was used for the logistic regression, with entry at p value of <0.05 and removal at p<0.1. Performance of the model was assessed with classification plots, Hosmer and Lemeshow test, concordance index, ROC analysis, and Nagelkerke R Square. Internal validation was conducted by assessing the predictive accuracy of the model on the second portion (30%) of the dataset, called the ‘validation dataset.’

### Ethical Approval

The NDNS survey was approved by the National Health Service Local Research Ethics Committees (LRECs) in the areas representing each of the 132 postcode sectors where fieldwork took place. The National Diet and Nutrition Survey (NDNS) was carried out by the Medical Research Council (MRC), Office for National Statistics, and the Dental Schools of the Universities of Birmingham, Newcastle, Dundee and Wales. Novel data analysis and interpretation was carried out by the authors independently of the funding sources based on the available dataset provided by the UK Data Archive. Written consent was obtained by the parents or guardians of children involved in the study. Appropriate permission was gained from the UK Data Archive to publish data from the NDNS.

## Results

Vitamin D levels were available for 1102 samples (mean = 62.1 nmol/L, 95%CI 60.4 to 63.7, SD = 27.7, skewness = 0.79). Of those samples, 35.1% were vitamin D insufficient.

Plasma Vitamin D levels decreased progressively with age in both sexes (table 1). There was no difference in the magnitude of this change between girls and boys. Univariate linear regression between Vitamin D blood levels and age was significant. There was no significant gender difference with linear regression.

**Table 1:** Vitamin D levels in relation to Age and Gender.

Sex	Age Group	25-hydroxyvitamin D levels (nmol/L)						
		Mean	Standard Deviation	Median	Percentile 05	Percentile 25	Percentile 75	Percentile 95
**Male**(51.7%)	**4–8 y**	75	29	71	32	54	95	128
	**9–13 y**	63	26	62	21	46	79	104
	**14–18 y**	55	26	51	18	35	68	101
	**Total**	63	28	61	22	45	79	117
**Female**(48.3%)	**4–8 y**	68	28	64	23	50	84	119
	**9–13 y**	61	25	60	21	44	74	106
	**14–18 y**	56	28	52	19	35	70	100
	**Total**	61	27	58	20	41	76	110

*Univariate linear regression between Vitamin D blood levels and: Age = r^2^ = 0.055, t −8.4, p<0.001, Sex: r^2^ = 0.063, t −1.5, p = 0.13. Differences in Mean between sexes; t-test; p = 0.48.*

*Age groups: 4–8 years (n = 285); 9–13 years (n = 423); 14–18 years (n = 394).*

Table 2 shows the candidate predictors for Vitamin D status and insufficiency. Serum vitamin D levels were strongly associated with ethnic group. Mean levels were double for white children (65 nmol/L) compared with non-white children (32 nmol/L). Children of South Asian ethnicity (n = 51) had a corrected (for age and season of measurement) mean vitamin D level of 22.2 nmol/L (95% CI 15.9–28.4 nmol/L) whereas the mean level for children of black ethnicity (n = 27) was 33.6 nmol/l (95% 25.0–42.2). Mean levels were approximately 40% higher for blood samples collected in June–November when compared with those taken December-May. Children whose families were on income support had significantly lower vitamin D levels. Children who spent more time doing outdoor exercise (at least half an hour/day/week) and less time watching TV per day (<2.5 hours) also had higher vitamin D levels. Vitamin D dietary intake had no influence on vitamin D status. However, children who were taking vitamin D supplements had higher overall vitamin D levels. Overweight and obese children had lower vitamin D levels. Children who lived in the North of England and Scotland (latitudes 53–59 degrees) also had lower levels than those living in other areas of the UK (p = 0.045; corrected for age, season, and ethnicity).

**Table 2:** Study population characteristics and associated vitamin D status.

Risk Factor	Characteristic	Mean (SD) 25(OH)D levels nmol/L	N (%) with <50 nmol/L 25(OH)D levels
**Ethnicity**	**White** (91%)	65.1 (26.5)	299/999 (30%)
	**Non-white** (9%)	32.2 (19.7)P<0.001	88/103 (85%)P<0.001
**Month Blood Taken**	**Dec-May**	51.1 (23.5)	266/503 (53%)
	**June–Nov**	71.2 (27.6)P<0.001	121/599 (20%P<0.001)
**Income Support**	**Yes** (12%)	56.2 (26.5)	66/135 (50%)
	**No** (88%)	62.9 (27.7)P<0.001	320/967 (33%)P<0.001
**Outdoor exercise and active play in a week**	**0–30 mins/day** (59%)	56.1	244/542 (45%)
	**30–60 mins/day**	65.4	53/189 (28%)
	**>60 mins/day**	69.6P<0.001	39/186 (21%)P<0.001
**Time spent watching TV in a week**(split in to approximately equal centiles)	**0–2.5 hours/day** (56%)	67.2	160/593 (27%)
	**>2.5 hours/day**	56.1	206/468 (44%)
	**0–2.5 hours/day** (56%)	67.2P<0.001	160/593 (27%)P<0.001
**Overweight or Obese (equivalent to BMI>25 and 30 respectively for adults; corrected for age and sex)**	Yes (22%)	63.1	98/247 (39%)
	No (78%)	58.2P = 0.01	289/852 (34%)P = 0.09
**Vitamin D Dietary Intake**(median = 2.2 mcg)	**<2.5 micrograms/day** (60% of samples)	61.9	205/614 (33%)
	**>2.5 micrograms/day** (40% of samples)	62.2*p = 0.8*	146/397 (37%)*p = 0.15*
**Taking Vitamin D containing Supplements**	**Yes** (8%)	68.2	25/85 (29%)
	**No** (91%)	61.5*p = 0.033*	360/1017 (35%)*p = 0.27*
**Region residing**	**North of England & Scotland; latitude = 53–59°** (n-365)	59.4 (95% CI 57.1 to 61.7)	
	**South of England & Wales; latitude = 50–53°** (n = 737)	62.3 (95% CI 60.7 to 64.0)*P = 0.045,* *(adjusted for age, month of blood intake, ethnicity, case weight)*	

Table 3 shows the logistic regression for the proposed predictors of VDI. Adolescents (14–18 years old) had an odds ratio (OR) more than three times that of younger children (4–8 years) of having VDI. Non-white children had 37 times the OR compared with white children of being vitamin D insufficient. There were marked seasonal effects, with vitamin D blood levels taken in the summer and autumn months higher (OR 6.5) than levels taken in the winter and spring months. The OR for children whose family were on income support was 2.2 times that of children not on support. Children who had less than half an hour outdoor exercise per day (OR 1.5) or watched more than 2.5 hours of TV/day (OR = 1.6) were more likely to have VDI. Overweight children had an OR 1.6 times higher of having VDI than that of normal and thin children. Finally, children not taking vitamin D supplementation had 3.7 times the odds of having VDI compared with those not taking supplementation. Table 4 sets out some example scenarios.

**Table 3:** Logistic Regression for proposed predictors of vitamin D Insufficiency with ‘training dataset’.

Predictors	Coefficient(log Odds)	WaldChi Square	Significance	Odds Ratio (OR)	95% C.I.for OR	
					Lower	Upper
**AGE Group (9–13 years)**	0.78	11.5	<0.001	2.29	1.39	3.44
**AGE Group (14–18 years)**	1.29	13.6	<0.001	3.62	1.83	7.18
**Non-white**	3.61	62.5	<0.001	36.8	15.1	89.9
**Blood Test taken Dec-May**	1.88	72.9	<0.001	6.54	4.25	10.1
**On Income Support**	0.80	7.53	0.006	2.22	1.26	3.92
**Not taking Vitamin D containing supplement**	1.30	6.99	0.008	3.66	1.40	9.58
**Watches more than 2.5 hours TV per day**	0.45	4.34	0.037	1.56	1.03	2.37
**Less than half hour exercise per day**	0.43	4.38	0.036	1.53	1.03	2.28
**Overweight**	0.45	3.52	0.059	1.57	0.98	2.50
**Constant**	−5.70	86.6	<0.001			

Nagelkerke R Square = 0.46.

Hosmer and Lemeshow test p = 0.45.

Comparators are: age (4–8 years); white; blood test taken June–November; not on income support; more than half hour exercise/day/week; less than 2.5 hours TV/day/week; taking Vitamin D supplements.

**Table 4:** Example scenarios based on model derived data.

Predicted risk of vitamin D insufficiency in:
A. non-white 7 year old child in the month of August:
1. not on income support; >half hour exercise per day; watches TV<2,5 hours/day; not overweight; taking vitamin d supplementation = **29% (95%CI 22–37%)**
2. on income support; <half hour exercise per day; watches TV>2,5 hours/day; overweight; not taking vitamin d supplementation = **93% (95%CI 87–96%)**
B. white 15 year old young person in the month of February:
1. not on income support; >half hour exercise per day; watches TV<2,5 hours/day; not overweight; taking vitamin d supplementation = **21% (95%CI 15–28%)**
2. on income support; <half hour exercise per day; watches TV>2,5 hours/day; overweight; not taking vitamin d supplementation = **89% (95%CI 79–95%)**
**A spreadsheet to calculate your own scenarios can be downloaded from** http://www.childdemyelination.org.uk/HealthProfessionals/vitamind/insufficiency.xls

The model had overall good performance as the Hosmer and Lemeshow goodness of fit test was non-significant (p = 0.45), the concordance (c) index was 0.8, and Nagelkerke R Square statistic was 0.46. ROC curve analysis of predicted probabilities showed that the model had good predictive accuracy with the area under the curve being 0.82 (95% CI 0.80–0.85 p<0.001). Validation statistics to assess the predictive accuracy of the original model developed showed that the model performed well, as at a cut off value of 0.5, 77% of VDI was correctly predicted (75% sensitivity, 79% specificity).

## Discussion

This is the first paediatric population based cross sectional study evaluating VDI in children with a wide age range (4–18 years) and with detailed information on: month of vitamin D measurement; latitude data; anthropometric measurements; exercise, dietary and socio-economic information. The results from this study confirm the high prevalence of VDI (35%) amongst UK children. Our findings also show that in children, there is no significant gender difference, but there is an increasing risk of VDI in older age groups. This may be because there are no recommendations for vitamin D supplementation in older children, but is unlikely, as in younger children although recommended supplement uptake is low. Older children may have less exposure to sunlight due to fewer incentives and opportunities to play outdoors. Supplements may increase vitamin D levels in older children, especially during the winter months given the higher risk of VDI shown (compared to those not on supplementation). The marked higher odds and difference in mean vitamin D levels demonstrated in our study for non-white children compared to white children, suggests that this group should be especially targeted in any preventative strategies. Although South Asian children had lower vitamin D levels than black children this did not reach significance levels, possibly due to the relatively small sample sizes. Additionally, children whose families were in receipt of income support had more than twice the odds of those not on income support; hence potentially government initiatives to tackle social inequalities may have an impact on vitamin D status. Marked seasonal differences found in our study also stress that preventative strategies if justified by the outcomes may be more necessary during the winter and spring months when vitamin D levels are at their lowest. The association of higher risk of VDI, and poorer vitamin D status amongst children who exercised outdoors less than half an hour/day/week, were overweight and watched more than 2.5 hours/day/week of TV highlights potentially modifiable risk factors. This implies that initiatives to increase safe sunlight exposure by increasing opportunities and encouraging more outdoor activities and play outdoors, particularly in the summer months, may have an impact on vitamin D status. This also suggests that guidelines encouraging safe sunlight exposure and how this message is portrayed may need to be revised. It is interesting that dietary intake was not associated with vitamin D status, and perhaps concerted efforts in this area would not be as effective as other suggested methods.

Strengths of this study include its design as a large population based survey with robust data collection. The logistic regression model has also been internally validated on a subset of the dataset. A limitation for this study is that this model was not externally validated in different populations or patient groups. Also sunscreen data was not available for assessment as a possible predictor. Another limitation is that the survey was set in 1998, and the prevalence of risk factors may have changed over the past decade. However this data is still likely to be valid and could be compared to future studies.

Prevention of VDI in childhood may potentially prevent longer term chronic disease with presentation in adulthood and this is an area which is under researched at present. Unfavourable cardiometabolic risk factors in adults, hyperglycaemia and the metabolic syndrome, type 1 diabetes, multiple sclerosis, malignancy and schizophrenia have all been implicated as potential consequences of early VDI as part of a complex interaction involving poor Vitamin D status [8], [14], [18], [27], [28]. The economic cost of prevention of poor vitamin D status in adults has also been reported, and provide evidence that preventative strategies are also cost-effective [29]. The model reported in this study may also inform the design of observational epidemiological studies when evaluating the impact and importance of vitamin D status on aetiology of immune mediated diseases such as multiple sclerosis and potentially the design of randomised control trials.

In conclusion, vitamin D insufficiency has a high prevalence in children, and clearer guidelines for its prevention including an increased awareness (especially in older children and at risk groups) based on these findings need to be devised and be more widely accessible. Our model needs to be externally validated in future studies to further explore its utility. Nevertheless, this study provides clinicians with some evidence to guide their clinical decisions, inform future public health preventative strategies, and help the design of future studies.
